# The Brain

**Published:** 1874-07

**Authors:** 


HALL'S

### JOURNAL OF HEALTH

AND

MISCELLANY.

VOL. XXI.—JULY, 1874.—NO. VII.

### THE BRAIN.

THE larger the brain in weight, in proportion to the weight of the body, the greater the intellect. Taking the average of a great many observations, the weight of the brain compared with that of the body is one to thirty-six; that is, if a man weighs 144 pounds, his brain would weigh four pounds, according to received estimates—in fish, one to five thousand; hence, in comparison to man, a fish is a fool. A comparative table would run thus: Man, 1 to 36; reptiles, 1 to 1,321; animals, 1 to 186; fish, 1 to 5,668; birds, 1 to 212.

The brain is divided mainly into two parts—the cerebrum, which is the brain proper, filling the front and top of the head, the intellectual part; the cerebellum, the hinder and lower portion of the skull, is the animal portion; it answers to that which is referred to as the back of the head, that portion above the nape of the neck.

If the whole brain is considered as two hundred parts the intellectual portion would be one hundred and seventy, the animal thirty. It will be readily seen, then, as phrenologists teach us, that the larger, the broader, the back part of the head is, between the ears, the more animal a man is, the more brutal in his nature, the more beastly his passions; but the higher the head at the top the more moral he is, and the broader the forehead the more intellectual. The heaviest brain ever weighed was that of Cuvier, the greatest naturalist who has ever lived; his whole life was devoted to the study of the nature of all animal and vegetable life. An idiot's brain would not weigh a third as much.

If a man has a very large brain he may be very great and very good—or a brutal monster, according to the relative quantities of the cerebellum and cerebrum.

| | | |
|---|---|---|
| Cuvier's brain weighed | 65 | ounces. |
| Daniel Webster's | 64 | " |
| Dr. Abercrombie's | 63 | " |
| Ruloff's | 59 | " |
| Average brain | 50 | " |
| Idiot's | 20 | " |

Every man has three natures—the intellectual, or broad forehead; the moral, or high head; the animal, or broad back head.

If a man has a high, broad forehead, flat top head and broad back head, he will have a great intellect, a brutal nature and a bad heart; such was Ruloff, the linguist, the burglar and wife and child murderer. These general truths of phrenology are undeniable.

One fifth of all the blood of the body goes to feed the brain; those who are starving soon go crazy. If, then, the brain uses so much of the blood of the body, the blood supplied to it must be good blood, or it cannot work well, cannot think well. Blood is bad when it is impure or thin, that is poor. Those who eat a great deal and exercise but little in the open air, or anywhere else, soon grow fat, and dull, and stupid; the blood is impure—has not had its impurities taken from it by the pure air going into the lungs to unload it; all know how "bad" the breath is as it comes direct from the lungs. If the blood is poor, is thin, has little nutriment in it, as in dyspeptic people, the brain is not properly nourished—does not think correctly; sometimes it seems to work not at all, and the nerves, which are its "wires," work badly, imperfectly, irregularly. Such persons are changeable, fretful, peevish, ill-natured, hysterical—smiling one minute, mad the next—forever complaining, never satisfied with anybody or with anything. Brain workers, then, of all men, ought to have pure, nourishing blood; to have this two things are always essential—an abundance of nutritious food and a large amount of exercise every day in the open air. The man who studies four hours of daylight, and spends the remainder of the daylight in interesting and exhilarating outdoor activities, will accomplish twice as much in a lifetime as he who spends eight hours in study, because the four hour student's brain being healthfully nourished, will work more vigorously, more clearly, more rapidly, and, above all, more energetically, and will be thinking from ten to fifteen years longer.

Something has been said of late about the brain requiring phosphorus for its proper nourishment, and that if persons feed on food which contains a larger amount of it than is common, they will think better, easier, more rapidly, more profoundly and more correctly. All this seems very "reasonable," but not a sufficient number of great men have arisen from fishing localities to have attracted any very special notice in this connection. As great men have lived hitherto as the world has any use for, we need not hope for any greater, and the greatest of these will be found generally to have lived pretty much upon what they could get. It follows, then, that there is phosphorus enough in the most ordinary kinds of human food; the point is to have a vigorous digestion of it; this supplies the "sinews of war." Here we find the fundamental requisite. The next thing is a pretty good sized head, well proportioned; these, combined with a good education and Christian principles, founded on solid Bible truth, will turn out as good, as great, as useful men as the world in its present constitution has any need for. The aim, then, should be in all cases to have a good sized head, a good digestion, a good education, and a good moral training. But observation the world over has shown that a moderate sized head, equally developed, other things being equal, makes the safest, best and most useful character.

A Pledget of Lint or Cotton moistened with a strong solution of saleratus, and applied to a boil or carbuncle in its forming stage, will check the disease and prevent suppuration. So says one who has tried it repeatedly.

